# New insights into the cardio-renal benefits of SGLT2 inhibitors and the coordinated role of miR-30 family

**DOI:** 10.1016/j.gendis.2023.101174

**Published:** 2023-11-23

**Authors:** Abdellatif El Khayari, Soukaina Miya Hakam, Gabriel Malka, Luc Rochette, Rachid El Fatimy

**Affiliations:** a Institute of Biological Sciences (ISSB-P), UM6P Faculty of Medical Sciences, Mohammed VI Polytechnic University (UM6P), Ben-Guerir 43150, Morocco; b Equipe d'Accueil (EA 7460): Physiopathologie et Epidémiologie Cérébro-Cardiovasculaires (PEC2), Université de Bourgogne – Franche Comté, Faculté des Sciences de Santé, 7 Bd Jeanne d'Arc, Dijon 21000, France

**Keywords:** Cardiac remodeling, Cardiovascular diseases, Endothelial dysfunction, Kidney, miR-30, SGLT2 inhibitor

## Abstract

Sodium-glucose co-transporter inhibitors (SGLTis) are the latest class of anti-hyperglycemic agents. In addition to inhibiting the absorption of glucose by the kidney causing glycosuria, these drugs also demonstrate cardio-renal benefits in diabetic subjects. miR-30 family, one of the most abundant microRNAs in the heart, has recently been linked to a setting of cardiovascular diseases and has been proposed as novel biomarkers in kidney dysfunctions as well; their expression is consistently dysregulated in a variety of cardio-renal dysfunctions. The mechanistic involvement and the potential interplay between miR-30 and SGLT2i effects have yet to be thoroughly elucidated. Recent research has stressed the relevance of this cluster of microRNAs as modulators of several pathological processes in the heart and kidneys, raising the possibility of these small ncRNAs playing a central role in various cardiovascular complications, notably, endothelial dysfunction and pathological remodeling. Here, we review current evidence supporting the pleiotropic effects of SGLT2is in cardiovascular and renal outcomes and investigate the link and the coordinated implication of the miR-30 family in endothelial dysfunction and cardiac remodeling. We also discuss the emerging role of circulating miR-30 as non-invasive biomarkers and attractive therapeutic targets for cardiovascular diseases and kidney diseases. Clinical evidence, as well as metabolic, cellular, and molecular aspects, are comprehensively covered.

## Introduction

Diabetes mellitus (DM), a group of metabolic disorders, is characterized by hyperglycemia, resulting from defects in insulin action, insulin secretion, or a combination of both. It is estimated to affect approximately 10.5% of the world population and is today considered a major health crisis. Diabetes can be classified into four types. Type 1 diabetes mellitus (T1DM), also called insulin-dependent diabetes mellitus, is an autoimmune disease in which insulin-producing pancreatic β-cells are destroyed, resulting in an absolute insulin deficiency.^1^ This form of diabetes accounts for 5%–10% of the overall diabetes patients and is most commonly seen in children and adolescents.^2^ Type 2 diabetes mellitus (T2DM) is the most common form of diabetes, accounting for 90% of all cases, and is commonly found in adults. T2DM is characterized by insulin resistance and insulin deficiency.^3^ Patients with T2DM are at a greater risk of developing cardiovascular diseases.

Over the past decade, with the prevalence of T2DM continuously increasing, improvement of the management of diabetic patients and diabetes-associated disorders, including chronic cardiovascular complications and nephrotic dysfunctions, has become an urgent need.^4^ Multiple classes of drugs controlling glycemia have quickly been developed and approved to meet this need, though, for diabetes, the holy grail in therapy remains diet and exercise. However, if normal glycemia cannot be achieved using these methods, insulin, and metformin are the first-line therapy.^4^ In addition to these antidiabetic therapies, sodium-glucose cotransporter inhibitors (SGLTis) are the most recent anti-hyperglycemic drugs.^5^ Contrary to the other approved therapies, sodium-glucose cotransporter 2 inhibitors (SGLT2is) act on the kidney to increase urinary glucose excretion, thereby lowering plasma glucose levels.^5^^,^^6^

To date, the available SGLT2is include empagliflozin (EMPA), canagliflozin (CANA), dapagliflozin (DAPA), sotagliflozin (SOTA), and ertugliflozin (ERTU). Their use in a large cohort has shown them to have several beneficial effects on T2DM patients. Administration of SGLT2is was associated with a significant reduction in all-cause mortality, as well as hospitalizations for events related to cardiac or renal complications.^7^, ^8^, ^9^, ^10^ The benefits produced by SGLT2is have drawn increased attention to their potential to protect the heart, vasculature, and renal function in people with or without diabetes.^11^^,^^12^

At the cellular level, given that SGLT2is are not expressed in the heart, the direct effect of these agents on myocardium is likely mediated by an off-target action; insights into their potential molecular targets are lacking. In this perspective, non-coding RNAs, particularly microRNAs, may play a key role in mediating or modulating the effect of SGLT2is and participating in their protective effect on the heart and kidneys.^13^^,^^14^

The microRNA-30 (miR-30) family, an important member of the microRNA family, is readily expressed in the heart.^15^ Just recently, its expression has been linked to several cardiac and renal pathologies, including myocardial infarction, heart failure (HF), acute coronary syndrome, cardiomyocyte ischemic injury, atherosclerosis, arrhythmias, fibrosis, and ventricular remodeling.^14^^,^^16^^,^^17^ The miR-30 family encompasses six microRNAs: miR-30a, miR-30b, miR-30c-1, miR-30c-2, miR-30d, and miR-30e.^15^ miR-30 family share a similar seed sequence at the 5′ end but differing compensatory sequences near their 3′ end.^18^ The variability in their non-seed region is a feature that allows the miR-30 family to target different genes, modulate their expression, and thereby regulate varying biological pathways.^19^ There is increasing evidence as to miR-30's involvement in a wide array of pathological and physiological states. The role and potential of these microRNAs as therapeutic targets remain to be investigated and are not yet clinically established despite accumulating data.

This review aims to summarize the current knowledge on the many beneficial effects of SGLT2is, with a particular focus on their protective outcomes on the heart and kidneys, in patients with T2DM. We aim to provide mechanistic insights linking the pharmacological effect to the clinically established outcomes. Furthermore, the potential of the miR-30 family as an emerging molecular biomarker in cardiovascular diseases and kidney diseases, as well as the possible interplay linking miR-30 dysregulation to SGLT2i-associated pathways are comprehensively discussed.

## SGLT inhibitors: metabolic, cardiovascular, and reno-protective outcomes in patients with or without diabetes

### SGLT1 and SGLT2 inhibitors in anti-diabetic therapy

Traditionally, insulin or insulin-dependent drugs have been used for the management of glycemic levels in diabetic patients. However, newer drugs such as SGLT2is, which are glucose-lowering agents, have emerged in the last decade, offering a newer approach by which to improve glycemic control/management in T2DM patients.^20^ The latter was first approved by the FDA in 2013 for use in adults with T2DM. SGLT2is work by reducing/preventing the reabsorption of glucose; the SGLT2, which is in the early proximal tubule is blocked, thus increasing urinary glucose excretion, and causing a decrease in the plasma glucose concentrations.^21^ The mode of action of SGLT2is is independent of the actions of insulin and is only dependent on the blood glucose levels.^20^^,^^22^ This mechanism of action gives SGLT2is several advantages in comparison to the other anti-hyperglycemic agents. Such advantages include no fatigue of the beta cells, reduced probability of hypoglycemia, a greater reduction in body weight and blood pressure, and an improvement in the lipid profile.^20^^,^^22^ CANA, DAPA, EMPA, ERTU, and SOTA are the five SGLT2is that have been approved to date by the FDA; they are now being considered as a second-line therapy for the management of T2DM. These drugs are widely used either as a monotherapy or in combination with other glucose-lowering agents for more effective management of glucose levels.^23^

CANA was initially approved by the FDA in 2013 for the management of diabetes. In 2017, DAPA became the first class of SGLT2i to be approved for use in China. Both CANA and DAPA are very effective in increasing glucosuria, thereby decreasing blood glucose levels when used either as a monotherapy or in combination. EMPA, the third agent in this class of drugs, has also been approved for use in the US and European markets. EMPA has proven its efficacy as an antidiabetic agent and resulted in an important decrease in hospitalizations from HF, death from cardiovascular disease, and clinically relevant renal events. ERTU has also been approved for use in both the US and European markets and is another SGLT2i used for the treatment of T2DM. Like the other three agents in this class, ERTU has demonstrated an effective reduction in blood pressure, body weight, and HbA1c.^24^ Lastly, SOTA, the most recently approved drug in this family, has previously demonstrated its ability to improve glycemic control when used as added to insulin therapy.^25^, ^26^, ^27^, ^28^ Functioning as a dual inhibitor of both SGLT1 and SGLT2, SOTA exhibits a significant capacity to augment glucose excretion in urine, concurrently lowering postprandial glucose levels. This effect is thought to be achieved through inhibition of the gastrointestinal SGLT1, which subsequently leads to the delayed absorption of glucose.^29^ Notably, the administration of SOTA in patients with T1DM has revealed a significant reduction in HbA1c levels, all without the unwanted consequence of weight gain.^25^^,^^30^ In addition to these, several similar drugs are commercialized in various countries, and many others are currently in clinical trials (*e.g.*, ipragliflozin, tofogliflozin, luseogliflozin, remogliflozin etabonate, ERTU).^31^

Emerging evidence suggests that this class of anti-hyperglycemic drugs may be administrated as first-line therapy in diabetic patients with other comorbidities, including diabetic cardiomyopathies, HF, nephrotic dysfunction, and non-alcoholic fatty liver disease.^32^, ^33^, ^34^, ^35^ Indeed, most observational studies into the benefits and risks of SGLT2is highlight their safety profile across diverse co-morbidities and therefore strongly recommend their use in patients harboring cardiac and nephrotic dysfunctions.^33^

In T1DM, insulin therapy is not without risk. This has pushed the need for the development of alternative therapies/drugs by which to improve glycemic levels, whilst reducing the risk of hypoglycemia. The beneficial impacts of SGLT2is in T2DM patients have led to a growing number of clinical studies investigating the latter's potential in T1DM subjects.^21^^,^^28^^,^^30^^,^^36^ Both DAPA and SOTA have been considered in the management of T1DM and have thus shown promising results in controlled clinical trials; the administration of these drugs resulted in a reduction in HbA1c without the increased risk of hypoglycemia.^30^^,^^37^ Moreover, two pivotal clinical trials, namely inTandem 1 (ClinicalTrials.gov, NCT02384941) and inTandem 2 (ClinicalTrials.gov, NCT02421510), have notably contributed to unraveling SOTA's efficacy and safety for patients with T1DM.^27^^,^^30^ These 52-week phase III trials have reported a statistically significant correlation between SOTA administration and several positive outcomes. These include reductions in HbA1c levels, a decrease in the occurrence of severe hypoglycemia, weight loss, lower insulin dose, and improved patient-reported outcomes.^25^^,^^28^ High-dose EMPA (10 or 25 mg per day) has also been tested as an add-on therapy to insulin for patients with T1DM. Data from these randomized, placebo-controlled trials have revealed improved glycemic control along with a clear beneficial effect on HbA1c, body weight, and total daily insulin.^38^^,^^39^ However, the use of SGLT2is in T1DM patients does come with an increased risk of diabetic ketoacidosis and mycotic infections.^40^ DAPA-assisted short-term therapy for T1DM does not increase the risk of diabetic ketoacidosis and has been approved by the European market in 2019 for use in T1DM adults presenting with a body mass index greater than 27 kg/m^2^.^41^ Nonetheless, diabetic ketoacidosis and euglycemic diabetic ketoacidosis associated with SGLT2is pose serious life-threatening complications, particularly in T1DM patients.^42^ Both reports and recommendations from clinicians and healthcare practitioners have underscored a compelling potential for preventing diabetic ketoacidosis. This preventive approach centers around patient education regarding SGLT2is' side effects, diligent ketone testing, and strict adherence to the sick day rule.^43^ Though current clinical trials do suggest a place for SGLT2is in the management of T1DM, longer clinical trials are required to determine what cohort of T1DM patients will benefit most from SGLT2i co-therapy and to establish the safety and persistence of their use in the longer term. To date, the use of SGLT2is in T1DM patients has yet to be approved by the FDA, who deems that the current benefits conferred by this therapy do not outweigh the risks that it carries.^44^^,^^45^

### Metabolic benefits: SGLT2 inhibitors induce a state of fasting mimicry promoting a systemic metabolic shift

**Figure 1:**
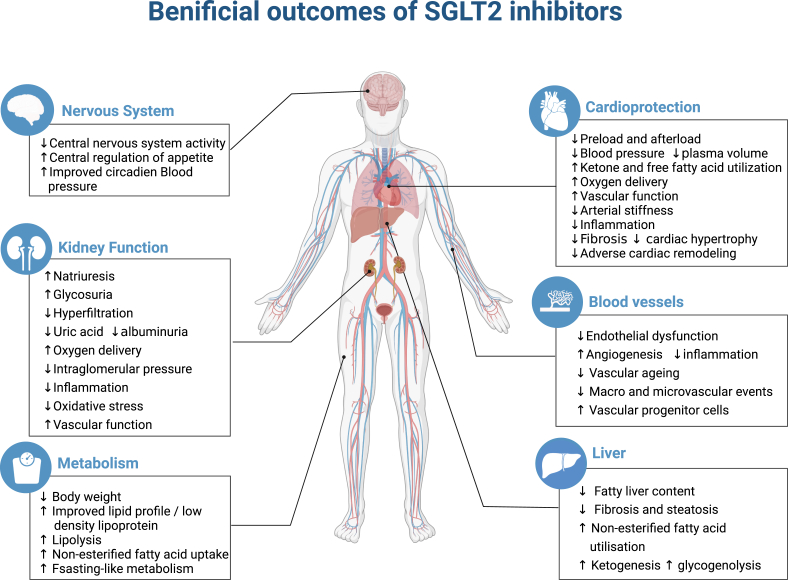
Summary of the various pleiotropic effects of SGLT2 inhibitors reported in diabetic and non-diabetic patients. Diverse favorable outcomes in patients with T2DM, as well as other cardio-renal disorders, were summarized. SGLT2 inhibitors impact the overall metabolism resulting in weight loss, improved lipid profile, and decreased obesity.

The use of SGLT2is in T2DM patients is now largely established, and clinical trials have demonstrated that their benefits go beyond that of glycemic control. One such benefit that has been largely studied is the metabolic outcomes associated with the use of SGLT2is. In addition to the improved glucose levels and decreased lipotoxicity conferred by SGLT2is, there is also an improvement in insulin resistance.^46^ The use of SGLT2is has also been associated with a decrease in serum uric levels, reduced visceral and subcutaneous fat masses, increased lipid utilization, and ketogenesis, thereby promoting weight loss and improving other associated cardiovascular risk factors.^22^^,^^47^ Research has also reported a change in protein catabolism and the utilization of amino acids as second energy substrates following the use of SGLT2is.^47^, ^48^, ^49^ SGLT2i therapy also decreased serum levels of total cholesterol and triglycerides and triglyceride accumulation in liver tissue; fecal cholesterol and triglyceride levels increased.^50^ Presently, there is still an ongoing debate regarding the changes in the serum high-density lipoprotein cholesterol and low-density lipoprotein cholesterol observed as a result of SGLT2i therapy^51^ (Fig. 1). Mechanistically, it has been proposed that treatment with SGLT2is triggers a chronic fasting-like state, enabling the installation of a new metabolic energy balance favoring ketone bodies and fatty acid utilization (lipid oxidation), over that of glucose.^22^^,^^52^^,^^53^ Besides the whole-body metabolism shifts, other evidence suggests a fuel selection mechanism in the heart, where β-hydroxybutyrate is freely taken up by the heart and oxidized in preference to fatty acids, resulting in reduced myocardial oxygen demand, and improved mitochondrial function. This process has been coined as the “thrifty substrate” theory by Ferrannini et al who sought to explain the benefits witnessed from the use of SGLT2is, notably the cardiovascular ones.^54^

In the heart, SGLT2is modulate the glucocentric metabolism in the failed heart and restore more efficient fuel energetics with increased oxidation of ketone bodies and branched-chain amino acids.^55^, ^56^, ^57^, ^58^ This metabolic reprogramming has been recently linked to a transcriptional paradigm featuring the activation of catabolic pathways accompanied by an increase of the hepatic and serum FGF21, a fasting-induced hepatokine that can eventually mediate the reduction of adipose tissue mass and lipolysis reprograming via sympathetic nervous system activation.^53^^,^^59^ SGLT2is also induce this state of fasting mimicry by down-regulating cholesterol, *de novo* lipogenesis, and steroid hormone pathways. The renin–angiotensin–aldosterone system was also found to be up-regulated.^52^^,^^53^^,^^60^ SGLT2is likely exhibit their metabolic effects by modulating multiple pathways to induce a shift in cellular reprogramming towards that of a dormancy state.^61^

### Clinically established cardio-renal benefits

Until recently, most clinical trials conducted have addressed the safety and cardiovascular benefits of SGLT2is. The multiple unexpected beneficial cardiovascular outcomes of EMPA, CANA, DAPA, SOTA, and ERTU have been consistently reported by completed randomized controlled trials including, but not limited to, EMPA-REG OUTCOME, CANVAS Program, DECLARE-TIMI, DAPA-HF, VERTIS CV, SOLOIST-WHF, SCORED, and SOTA-P-CARDIA trials.^62^ Such benefits include a decrease in deaths from cardiovascular causes, a reduction in the relative risk of HF and hypertensive HF, and hospitalization from these, as well as a remarkable decrease in all-cause mortality.^62^^,^^63^ While these cardiovascular benefits were initially observed in clinical trials involving patients with T2DM or T1DM, recent data suggest that these advantages might extend beyond diabetic status.^12^^,^^64^ Emerging evidence indicates that SGLT2is hold potential cardio-renal benefits for non-diabetic patients. Notably, SOTA has recently gained FDA approval for treating HF, regardless of diabetic status or left ventricular ejection fraction.^65^ In addition, an ongoing clinical study is currently examining SOTA's cardiovascular effects and mechanisms in non-diabetic patients with HF and preserved ejection fraction.^64^ Noteworthy, completed clinical trials involving SOTA have reported a significant reduction in cardiovascular outcomes among diabetic patients and those experiencing a recent worsening of HF (SOLOIST-WHF).^66^ Additionally, the SCORED trial has shown that patients with T2DM and moderate renal impairment experience reduced risk of death from cardiovascular causes, HF hospitalizations, and urgent hospital visits due to HF.^66^

Diabetic kidney disease develops in approximately 30%–50% of diabetic patients. Randomized controlled trial data showed significant improvement in prognosis in patients with and without diabetes.^67^^,^^68^ Two clinical trials CREDENCE and DAPA-CKD both demonstrated the protective effects of DAPA and CANA respectively on kidney function and chronic kidney disease progression. SGLT2i administration was correlated with a reduction in death from kidney disease, risk of kidney failure, and sustained decline in the estimated glomerular filtration rate.^62^^,^^63^ Similarly, evidence from other clinical studies including EMPA-CKD trial, CANVAS program, and DECLARE-TIMI have reported significantly reduced risk of dialysis and transplantation and protection from acute kidney injury in T2DM subjects treated with EMPA.^69^ SGLT2is appear to exert multiple favorable cardiovascular and renal benefits, including attenuation of adverse cardiac remodeling (CR), improvement of atherosclerotic and fibrotic markers, as well as improvement of endothelial and vascular functions.^63^ In the kidneys, SGLT2is slow the loss of glomerular filtration rate, increase uric acid clearance, alleviate fibrosis, and limit glomerular injury.^62^^,^^70^^,^^71^

Of note, the effects of SGLT2is on cardiovascular and renal outcomes are intertwined. It is generally accepted that improvement of cardiovascular outcomes further ameliorates and preserves kidney function via a bidirectional interaction involving hemodynamic impact and renin–angiotensin–aldosterone system with the restoration of tubuloglomerular feedback.^72^ Furthermore, the sympathoinhibitory effect of SGLT2is resulted in another hypothesis linking the SGLT2 inhibition in the proximal tubule with the observed beneficial cardiovascular outcomes. It has been postulated that SGLT2is exhibit their protective effects in the context of HF by reducing the central sympathetic output through direct inhibition of the renal stress^73^ (Fig. 1).

## General overview of circulating miR-30 family as candidate biomarkers in cardiovascular and renal diseases

### miR-30 family in heart and vascular diseases

The biological functions of the miR-30 family have been clearly identified and have potential roles in widespread physiological and pathological states; recently, it has been identified as one of the microRNAs linked to cardiovascular pathologies. This family of microRNAs is highly expressed in the heart, and their dysregulation has been associated with several cardiovascular dysfunctions, notably HF, acute coronary syndrome, cardiomyocyte ischemic injury, atherosclerosis, arrhythmias, fibrosis, and ventricular remodeling.^15^^,^^16^^,^^74^, ^75^, ^76^, ^77^, ^78^, ^79^, ^80^, ^81^

At present, the dysregulation and the implication of the miR-30 family in heart diseases are being increasingly evidenced. Thus, their potential as diagnostic and prognostic biomarkers in a variety of pathological states was consistently highlighted. Indeed, microRNA profiling in HF patients has revealed abnormal expression of miR-30. It was significantly down-regulated in HF patients in contrast with healthy patients.^76^^,^^82^ Accordingly, a one-year follow-up study involving 96 patients clinically diagnosed with acute HF showed that low serum miR-30d level was correlated with poor survival of those patients.^75^ This study emphasized the predictive value of circulating miR-30 in acute HF patients. The potential of miR-30d as a circulating biomarker of CR has been further evidenced by quantification of miR-30d levels in plasma extracellular vesicles of mice and humans with HF, which demonstrated a significantly lowered expression associated with adverse CR in HF patients.^83^ Low expression of miR-30e has also been connected to dilated cardiomyopathy and aortic stenosis.^84^ miR-30 expression is correlated with collagen volume fraction, a key marker of cardiac fibrosis.^85^^,^^86^ Additionally, subsequent studies in various animal models have shown that miR-30 dysregulation is common in atrial fibrillation and may play a key role in structural and electrical CR.^80^^,^^81^^,^^87^

Conversely, other clinical and animal studies have reported up-regulation of miR-30 in myocardial infarction, left ventricular dysfunctions, and HF.^88^, ^89^, ^90^ Plasma level of miR-30 was significantly higher in patients with non-ischemic HF than that of control patients or patients with left ventricular hypertrophy.^88^^,^^90^ Yet, miR-30a-5p is elevated in the serum and plasma of acute myocardial infarction patients who developed left ventricular dysfunction and HF. This study emphasized the prognostic value of circulating miR-30 as a biomarker of left ventricular dysfunction.^89^ Furthermore, Shen and colleagues demonstrated that the expression of miR-30 is up-regulated in myocardial infarction mouse models and hypoxic cardiomyocytes; the authors reported a key role for miR-30 in modulating cardiomyocyte injury and highlighted its therapeutic potential for ischemic heart disease.^78^

miR-30 level is also substantially lower in hypertension patients than in healthy controls and was negatively correlated with increased carotid intima-media thickness, a well-known surrogate marker for atherosclerosis.^79^

Interestingly, a translational pilot study focusing on response to cardiac resynchronization therapy (CRT) in HF patients with dyssynchrony found that miR-30d is ultimately associated with positive CRT response and that this microRNA has a novel functional role in CR.^91^ An additional prospective study enrolling 81 patients with HF has demonstrated that those patients who responded to CRT were characterized by an up-regulation of miR-30e. In addition, data showed that the expression of both miR-30d and miR-33e increased after one year of CRT.^92^

Altogether, these findings suggest that miR-30 family members are involved in the pathological processes of a variety of heart and vascular disorders, as well as in the responsiveness to conventional therapy. Their potential as non-invasive biomarkers is undoubtedly of great interest.

### miR-30 in kidney diseases

Among other microRNAs, the members of the miR-30 family, in particular, are closely related to several kidney diseases, including diabetic kidney dysfunction, renal fibrosis, and focal segmental glomerulosclerosis.^93^ Contrary to the heart, in the kidney, the expression of miR-30 family members is lower. For instance, evaluation of urinary exosomal microRNA expression in subjects with T2DM and diabetic kidney disease demonstrated that the expression profile of miR-30b-5p was consistently altered as a consequence of renal dysfunction.^17^ Patients with diabetic kidney diseases were seen to have reduced miR-30e-5p plasma and urine levels, as reported by Dieter et al in 2019^94^; to date, it is known that miR-30 protects against both inflammation and fibrosis.^95^ In podocytes, the miR-30 family, namely miR-30c-1, miR-30b, miR-30d, and miR-30c-2 were reported to target four functional genes involved in podocyte apoptosis (RAGE and IRR3) and cytoskeletal arrangement (VIM and HSP20). This further supports the functional role of miR-3O in podocytopathies and therefore in kidney dysfunctions.^96^

Acute kidney injury, sometimes referred to as acute renal failure, refers to a sudden decline in the ability of the kidneys to perform their normal functions. This condition is usually the result of a serious underlying illness, such as ischemia, sepsis, nephrotoxicity, bladder, and urinary outflow obstruction.^97^ microRNAs' role in acute kidney injury was first reported in 2010 by Wei et al.^98^ Since then, three miR-30 family members, namely miR-30a, miR-30c, and miR-30e have been proposed as accurate early predictive biomarkers of kidney injury after contrast administration.^99^ In a 2017 study, Du et al reported that miR-30c may be a potential player in minimizing cisplatin-induced nephrotoxicity, after reporting that the latter regulates renal tubular cell apoptosis. They also noted that all of the miR-30s were significantly down-regulated in models of cisplatin-induced apoptosis both *in vivo* and *in vitro*.^100^

The hallmark of chronic kidney disease is renal fibrosis, which is usually the main cause of end-stage renal disease.^101^ Several studies have shown that the miR-30 family plays an important role in the process of renal fibrosis. In 2020, Yuan et al showed that miR-30c, which is involved in the regulation of several pathways in diabetic nephropathy, inhibits renal fibrosis in the latter, by decreasing ROCK2.^101^ The miR-30 family is also involved in the process of renal fibrosis through the regulation of mitochondrial ATP and connective tissue growth factor production as well as inhibition of epithelial-to-mesenchymal transition.^102^ Furthermore, miR-30a suppresses the expression of snail family zinc finger 1, a potent transcriptional factor mediating epithelial-to-mesenchymal transition in renal tubular epithelial cells.^103^

Many studies on miR-30 have also decisively demonstrated the involvement of this family in podocyte homeostasis and injury. Focal segmental glomerulosclerosis is a condition in which scar tissue develops on the glomeruli of the kidney and is often caused by a variety of diseases, notably diabetes, obesity, and kidney diseases.^104^ Patients presenting with focal segmental glomerulosclerosis are also seen to have reduced levels of miR-30 in the glomerulus; the latter is usually found in abundant levels in the kidney. Such is important in the context that *in vitro* sustenance of miR-30 levels offers protection to podocytes against TGF-B-induced injuries; TGF-B is a regulator of kidney pathogenesis, including induction and acceleration of pro-fibrotic response.^105^ In addition, Liu et al also reported that miR-30d was negatively regulated by TGF-B, which was done through the Smad signaling pathway.^104^ TGF-β represses miR-30d through a repression complex composed of three factors, Smad2/3, HDAC3, and NCoR, thereby promoting podocyte damage and apoptosis.^104^ In line with this, exogenous expression of miR-30 was found to reduce PAN or TGF-β-induced podocyte injury *in vitro* and *in vivo*. miR-30 prevents podocyte injury by reducing apoptosis and cytoskeletal damages via targeting Notch1 and p53 signaling.^93^

## miR-30 family and SGLT2 inhibitors: versatile players in endothelial physiology and dysfunction

**Figure 2:**
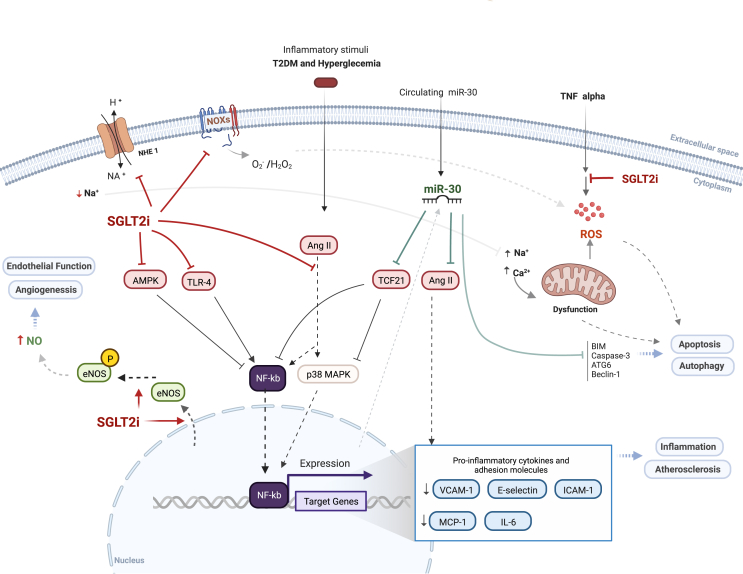
Potential mechanisms involved in SGLT2 inhibitor- and miR-30-mediated effects on endothelial cells and endothelial dysfunction. According to the reviewed literature, the mechanisms underlying the effect of SGLT2 inhibitors and miR-30 on the endothelium are mainly mediated by a potent action on multiple master regulators such as TCF21, TLR-4, AMPK, and angiopoietin 2, leading to inhibition of NF-κB and reduced expression of proinflammatory cytokines and adhesion molecules. In addition, miR-30 and SGLT2is act indirectly by attenuating oxidative stress and improving endothelial cell survival. SGLT2, sodium-glucose cotransporter 2; T2DM, type 2 diabetes mellitus; NHE1, Na^+^/H^+^ exchanger 1; TCF21, transcription factor 21; TLR-4, Toll-like receptor 4; AMPK, AMP-activated protein kinase; NF-κB, nuclear factor-κB; eNOS, endothelial nitric oxide synthase; NO, nitric oxide; NOXs, NADPH oxidases; TNF, tumor necrosis factor; ROS, reactive oxygen species; Ang II, angiotensin II; ATG6, autophagy-associated protein 6; V, vascular cell adhesion molecule-1; ICAM-1, intercellular adhesion molecule-1; MCP-1, monocyte chemoattractant protein-1; IL-6, interleukin-6.

A series of basic and clinical studies have emphasized the importance of endothelial dysfunction as a key factor and relevant biomarker associated with the initiation and progression of vascular diseases in diabetic patients.^106^ In line with this, several *in vitro* and *in vivo* investigations have attempted to explain the protective effect of SGLT2is on endothelial function in the heart and kidneys.^107^ Several mechanisms have been proposed thus far, and increasing data are supporting the anti-inflammatory, angiogenic, and atheroprotective outcome of SGLT2 inhibition.^108^ In recent years, a number of transcriptomic investigations and mechanistic studies have explored the role of microRNAs in regulating endothelial cells' function. Current evidence reports the implication of the miR-30 family in endothelial cell autophagy, angiogenesis, and inflammatory process in a variety of pathologic conditions including atherosclerosis and CR (Fig. 2).

In the endothelium, the ectopic expression of miR-30 was found to significantly improve endothelial dysfunction by mediating an anti-inflammatory effect. According to Demolli et al, the miR-30-5p family acts in an anti-inflammatory manner in endothelial cells by repressing the expression of angiopoietin 2, a pro-inflammatory protein, and inflammatory adhesion molecules such as vascular cell adhesion molecule-1, intercellular adhesion molecule-1, and E-selectin.^109^, ^110^, ^111^ The authors reported that laminar shear stress and overexpression of Krüppel-like factor 2 up-regulate miR-30 family members' expression in endothelial cells. Furthermore, miR-30-3p was seen to reduce inflammation and improve endothelial cell injury *in vitro* through direct targeting of transcription factor 21 and suppression of NF-κB (nuclear factor-κB) signaling and mitogen-activated protein kinase pathway.^112^ Subsequently, it is suggested that miR-30 may suppress the progression of atherosclerosis via its anti-inflammatory effect in the endothelium.^109^^,^^111^, ^112^, ^113^

In addition to inflammation, the implication of miR-30 in atherosclerotic diseases has also been associated with a pivotal role in endothelial cell autophagy, apoptosis, oxidative stress, and angiogenesis. For instance, in autophagy, the related research has evidenced that miR-30 up-regulation was responsible for suppressing the translation of autophagy-associated protein 6 mRNA, decreasing its protein level, thus impairing endothelial cell autophagy, and protecting against the development of atherosclerosis.^114^ Another study has demonstrated that miR-30a can attenuate endothelial cell autophagy via translational control of another autophagy-related protein, Beclin-1.^115^ Moreover, miR-30d was shown to inhibit cell autophagy by directly acting on TG5, phosphoinositide 3-kinase, and Beclin1.^116^ Although the same study reported miR-30d-mediated increased apoptosis, evidence showed that miR-30 acts in an anti-apoptotic fashion in endothelial cells by modulating the expression of several apoptotic regulators such as BIM and caspase-3 and by reducing oxidative stress.^111^^,^^117^, ^118^, ^119^ Of note, upon endothelial injury, it is known that endothelial cells activate autophagic machinery to enable cell homeostasis and maintain cell viability. However, elevated autophagy could lead to autophagy-associated cell death (Autosis).^114^ Likewise, apoptosis is activated under injury conditions and is thought to contribute to endothelial dysfunction progression. Therefore, the miR-30 family seems critical in autophagy and apoptosis turnover.

One of the most studied aspects regarding the effect of SGLT2is on endothelial dysfunction lies in its systemic hypoglycemic effect. A number of *in vivo* studies including diabetic and non-diabetic animal models have been used to explore the effect of SGLT2is on endothelial function.^108^^,^^120^ They were proven to contract lipopolysaccharide- and TNFα-induced inflammation, and thereby regulate vascular endothelial cells' activation, with a significant reduction in pro-inflammatory cytokines and adhesion molecules.^108^^,^^121^ Mechanistically, it has been reported that CANA reduced AMP-activated protein kinase signaling in human coronary artery endothelial cells. AMP-activated protein kinase is known to repress NF-κB and negatively control the expression and the secretion of pro-inflammatory chemokine/cytokines including interleukin-6, monocyte chemoattractant protein-1, and vascular cell adhesion molecule-1.^122^^,^^123^ Most recently, it has been shown that the anti-inflammatory effect of the SGLT2i DAPA was mediated by repressing Toll-like receptor 4 and p-NF-κB p65 expression in human umbilical vein endothelial cells.^121^ Accordingly, using apoE mice, Ortega and colleagues have demonstrated that EMPA inhibits p38 mitogen-activated protein kinase pathway and p-NF-κB p65 signaling which are known to be associated with cell activation and secretion of pro-inflammatory molecules.^124^ A proteomic study has further demonstrated the association of EMPA with the reduction of inflammatory biomarkers in patients with HF and preserved ejection fraction.^49^

In addition to vascular inflammation, oxidative stress is also implicated in endothelial balance. Indeed, increased intracellular reactive oxygen species is a key hallmark of endothelial dysfunction. The SGLTi-mediated protective effect has been directly linked to the reduction of reactive oxygen species levels and restoration of nitric oxide bioavailability in vascular endothelial cells.^125^^,^^126^ While enhanced nitric oxide bioavailability was initially attributed to up-regulation of endothelial nitric oxide synthase (eNOS) expression and phosphorylation, recent data suggested an eNOS-independent mechanism.^126^ The involvement of Na^+^/H^+^ exchanger 1 (NHE1) and NADPH oxidases in SGLT2i-mediated anti-oxidative stress in endothelial cells has been extensively reported.^107^^,^^127^ Furthermore, SGLTis have been shown to mitigate oxidative damage under a hyperglycemic state through amelioration of the impaired phosphorylation of AKT and eNOS^ser1177^, and thereby improve endothelial functioning and vascular homeostasis.^6^^,^^120^^,^^128^

Beyond this, the contribution of ferroptosis to the pathogenesis and progression of endothelial dysfunction has been reported.^129^ SGLT2is, notably EMPA and CANA, are shown to prevent ferroptosis and hence improve cardiovascular outcomes in non-diabetic mice and rat models of HF with preserved ejection fraction.^130^^,^^131^ The mechanisms underlying their regulatory effect on ferroptosis have generally been linked to the restoration of iron metabolism and reduction of lipid peroxidation.^130^

At present, increasing research is being directed towards investigating new mediators of endothelial dysfunction, such as microRNAs, lncRNAs, and circRNAs.^132^^,^^133^ For instance, Zeng et al demonstrated that by silencing a circRNA, namely, circ_0001879, they were able to inhibit the proliferation and migration of human retinal microvascular endothelial cells under high-glucose conditions, via modulating miR-30-3p expression; this member of miR-30 family is associated with reducing inflammatory markers and improving endothelial dysfunction.^134^

According to the above evidence, SGLT2is and the miR-30 family most likely play a key role in regulating endothelial cells' function and ultimately participate in the onset of endothelial dysfunction, through divergent mechanisms. Owing to their mutual effects on inflammation, angiogenesis, and survival of endothelial cells, and given the illusive mechanisms underlying SGLT2i effects, an interplay between both actors at the cellular level is highly possible. miR-30 family may be involved in mediating SGLT2is' effect at the epigenetic level in endothelial cells in a synchronous manner. Furthermore, the synergetic effect between them could be investigated in pathological conditions to explain the mechanistic relationship and the possible interplay.

## Role in preventing adverse cardiac events and pathological remodeling

Pathological CR is a leading cause of HF and mortality worldwide. This condition, which is defined as a group of molecular, interstitial, and cellular changes, is a deleterious progressive process, that clinically manifests as changes in size, shape, and function of the heart as a result of cardiac injury, notably myocardial infarction.^135^ CR is a reprogramming process involving gene expression alterations in the myocardium, where epigenetic regulations, particularly through microRNAs, seem to be crucial in mediating transcriptomic and translational changes.^13^ CR is currently regarded as a complex condition involving a set of altered physiological and cellular pathways ranging from cardiac fibrosis, myocardial apoptosis, chronic inflammation, angiogenesis, and arrhythmia.^136^, ^137^, ^138^

It is widely suggested that CR is a key process mediating the observed clinical benefits of SGLT2is. As stated above, the implication of SGLT2is as well as the miR-30 family in CR-related mechanisms, particularly, cardiac fibrosis, cardiomyocyte apoptosis, and inflammation, have been extensively reported during recent years. Therefore, it is now clear that both actors exhibit a potential role in CR and could be considered as new patterns that may hold hope for novel therapeutic strategies to slow, arrest, or even reverse the remodeling process in patients with heart disease.

### SGLT2 inhibitors attenuate reverse cardiac remodeling: basic and clinical evidence

Evidence regarding whether SGLT2is attenuate adverse CR and improve the prognosis of patients with failing hearts is being increasingly investigated.^63^ Numerous clinical trials have explored the beneficial effects of SGLT2is on the heart and the management of its pathological remodeling. It has been shown that EMPA treatment reduced left ventricular volume and showed improvement of left ventricular functional capacity among non-diabetic patients with HF and preserved ejection fraction (EMPA-TROPISM; NCT03485222),^139^ as well as patients with both HF/preserved ejection fraction and T2DM (SUGAR-DM-HF; NCT03485092). The data from this trial highlighted the concept of reverse CR as a plausible mechanism underlying the cardiac benefits associated with SGLT2is.^140^ Using cardiac magnetic resonance, the EMPA-TROPISM study has found that regardless of the diabetic status of the patients, EMPA mediates regression of interstitial myocardial fibrosis in HF with preserved ejection fraction.^49^ On the other hand, the Empire-HF study (NCT03198585) showed that EMPA was associated with a modest but significant reduction in left ventricular and left atrial volumes; however, there was no association with ejection fraction reported by this study.^141^ Moreover, the EMPA-HEART study (NCT02998970), a phase IV double-blind, randomized, placebo-controlled trial has also assessed the effect of EMPA on cardiac structure and function in patients with T2DM. Data from this clinical investigation illustrated that EMPA reduced extracellular myocardial volume in contrast to the placebo. The study revealed significant differences in extracellular compartment volume, indexed-extracellular compartment volume, and left ventricular mass indexed to body surface area.^142^ In support of this, other investigations have shown that short-term EMPA treatment significantly regresses left ventricular mass, promotes reverse remodeling, and improves diastolic function in diabetic patients with cardiovascular diseases.^143^ Additional clinical trials are presently being conducted, and the outcomes of these investigations may help to thoroughly establish the influence of SGLT2is on CR. For instance, the CARDIA-STIFF study (NCT04739215) aims to investigate the underlying mechanisms behind SGLT2i-associated effects on patients with HF and preserved ejection fraction. This study will ultimately investigate the impact on myocardial remodeling by monitoring mass, volume, and ventricular function using cardiac magnetic resonance. The correlation of myocardial remodeling with intrinsic diastolic properties is also one of the objectives of this clinical trial. Similarly, another phase IV study launched in 2021: PRESTIGE-AMI (NCT04899479) is investigating the effectiveness of SGLT2is in reducing the size of infarction and myocardial remodeling in patients with acute myocardial infarction and high risk of HF using cardiac magnetic resonance. Of note, previous research has reported an association between EMPA and improvement of ischemia-reperfusion injury, reduction in myocardial infarction size, and microvascular obstruction, in a porcine model.^128^ Cardiac magnetic resonance is also being used in the SOTA-P-CARDIA study (NCT05562063) to investigate the adverse CR of SOTA in non-diabetic HF patients. This ongoing study focuses primarily on the changes in left ventricular mass, myocardial mechanics, and interstitial myocardial fibrosis. It aims also to investigate SOTA's impact on exercise capacity and quality of life for a period of six months.^64^ Another promising study is the EMPACT-MI trial, which explores the efficacy and safety of EMPA in post-myocardial infarction patients by assessing the time to first hospitalization for HF or all-cause mortality, as a primary end-point.^144^

Many mechanisms have been suggested to explain SGLT2i-mediated reverse CR. Notably, the anti-fibrotic effect is largely evidenced, showing a significant impact on pathological remodeling.^145^, ^146^, ^147^ EMPA has been reported to reduce cardiac fibrosis through modulation of myofibroblast function, and suppression of the expression of several pro-fibrotic factors, including collagen type I α1, actin alpha 2 (smooth muscle), connective tissue growth factor, fibronectin 1, and matrix metalloproteinase 2.^147^ Similar findings from non-human studies have reported a remarkable reduction in myocardial interstitial fibrosis after EMPA treatment.^148^, ^149^, ^150^ Furthermore, other *in vivo* studies with DAPA have suggested that DAPA alleviates cardiac fibrosis by targeting mitogen-activated protein kinases.^145^^,^^146^^,^^151^ For instance, using transverse aortic constriction mice, Shi et al have found that DAPA inhibits JNK, p38, and FoxO1 signaling pathways, which was associated with the reduction of myocardial interstitial and perivascular fibrosis. The down-regulation of mitogen-activated protein kinase/activator protein-1 signaling by DAPA has been found to be NHE1-dependent.^151^ Moreover, DAPA protected against myocardial fibrosis by inhibiting resident fibroblast activation, collagen secretion, and improvement of cardiac dysfunction. Mechanisms behind these changes were found to be related to suppression of EndMT and AMP-activated protein kinase α-mediated inhibition of TGF-β/Smad signaling.^145^

Another direct target of SGLT2is is NHE1. Numerous studies evidenced that EMPA (and other SGLT2is) directly inhibit NHE1 activity, which is reported to mitigate the development of fibrosis, hypertrophy, and worsening of HF.^152^, ^153^, ^154^ However, evidence concerning NHE inhibition is marked by significant divergence, and a lack of consistency across multiple studies conducted on cardiomyocytes and isolated hearts. Chung and colleagues, for instance, reported that EMPA does not affect cardiac NHE activity or intracellular Na^+^ levels in isolated rat ventricular cardiomyocytes.^155^ Nevertheless, most studies align in supporting the NHE-1 inhibition as a direct target of SGLT2is in different cardiac cells, including cardiomyocytes, endothelial cells, and cardiofibroblasts.^22^^,^^156^ The divergent outcomes reported by some researchers could be attributed to the multiple methodological differences between these studies. Furthermore, DAPA attenuated abnormal remodeling in diabetic hearts by reducing the activation of Nlrp3/ASC inflammasome and fibrosis.^157^

**Figure 3:**
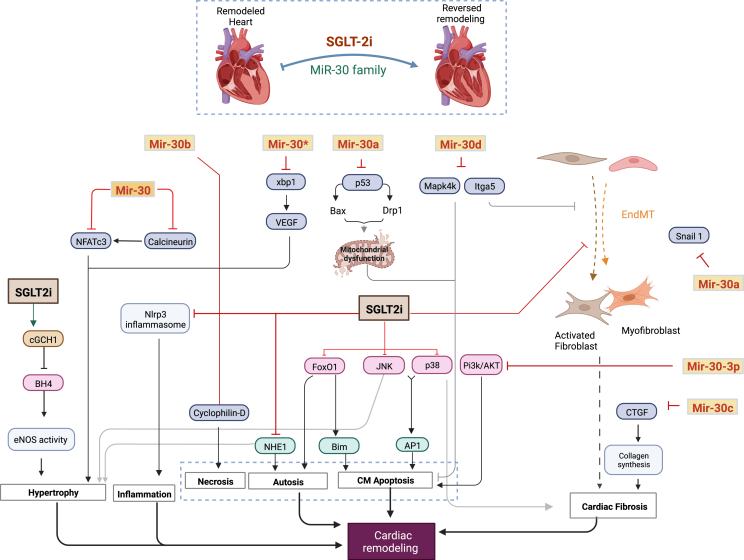
Coordinated effects of miR-30 family and SGLT2 inhibitors on reversing cardiac remodeling. SGLT2 inhibitors and miR-30 family together, have proven to regulate many cellular and pathological processes involved in cardiac remodeling. The current literature supports a set of common mechanisms explaining their combined salutary effects in this context, which are most likely related to cardiomyocyte cell death via a protective effect against induced apoptosis and necrosis, and to the amelioration of cardiac fibrosis and hypertrophy. SGLT2, sodium-glucose cotransporter 2; NHE1, Na^+^/H^+^ exchanger 1; AP1, activator protein 1; cGCH1, cardiac GTP enzyme cyclohydrolase 1; BH4, tetrahydrobiopterin; eNOS, endothelial nitric oxide synthase; xbp1, X-box binding protein 1; VEGF, vascular endothelial growth factor; Drp1, dynamin-related protein-1; Itga5, integrin α5; Snail 1, snail family zinc finger 1; CTGF, connective tissue growth factor; CM, cardiomyocyte.

In addition to their anti-fibrotic action, SGLT2is reduce myocardial remodeling by improving cardiac energetics and enhancing mitochondrial function and biogenesis.^57^^,^^158^^,^^159^ Using the porcine model, one recent study showed that EMPA ameliorates left ventricular remodeling and left ventricular systolic function by shifting myocardial metabolism energetics from glucose to enhanced utilization of ketone bodies, free fatty acids, and branched-chain amino acids.^57^ In support of this, Shao et al reported similar effects of EMPA on T2DM rats. EMPA was found to improve atrial structural and electrical remodeling by mitigating metabolic abnormalities in mitochondrial bioenergetics^158^ (Fig. 3).

### miR-30 family: a key mediator of hypertrophy, cardiomyocyte apoptosis, and fibrosis

The effect of the miR-30 family on SGLT2i-mediated cardiac outcomes is still not well understood. However, an accumulating number of studies continue to investigate the implication of this microRNA in CR. To date, the abnormal expression of miR-30 has been directly linked to ventricular hypertrophy in the human pathologic heart.^160^, ^161^, ^162^ Dual and colleagues reported that reduced miR-30 expression in the early phase of cardiac hypertrophic animal models and human failing hearts is closely associated with the up-regulation of X-box binding protein 1 and its downstream target gene vascular endothelial growth factor.^163^ The study demonstrated that the miR-30∗ (miR-30-3p) family directly targets X-box binding protein 1 in cardiomyocytes, thereby attenuating the expression of cardiac vascular endothelial growth factor. Of note, vascular endothelial growth factor along with X-box binding protein 1, are known to play a significant role in autophagy, cardiac angiogenic imbalance, and progression of cardiac hypertrophy.^163^^,^^164^ Furthermore, genetic silencing of miR-30 in cardiomyocytes was found to lead to cardiac hypertrophy. This *in vitro* and *in vivo* study also showed that miR-30 supplementation was able to attenuate/inhibit chronic kidney disease-induced left ventricular hypertrophy through up-regulation of calcineurin/NFATc3 signaling.^161^ Thus, it is suggested that miR-30 is key in cardiac hypertrophy and may represent a potential therapeutic target to mitigate myocardial remodeling.^16^

Additionally, atypical expression of miR-30 in failing hearts contributes to CR via induction and promotion of myocardial fibrosis. In the kidney, miR-30 level is inversely correlated to several fibrotic mediators in the myocardium and is thought to play a crucial role in collagen synthesis, fibroblast activation, and matrix remodeling, in particular, T-cell growth factor, snail family zinc finger 1, and integrin α5 in hypertrophic heart.^80^^,^^81^^,^^83^ The decreased expression of miR-30 in the fibrotic heart is associated with the up-regulation of these profibrotic proteins. Rudy and colleagues (2009) have previously reported that knockdown of miR30 increased connective tissue growth factor levels in cultured cardiomyocytes, resulting in enhanced production of collagens.^81^ In cardiac fibroblasts, miR-30a was also found to directly target Snail1, and alter the expression of periostin, both of which are associated with myocardial fibrosis.^83^ miR-30d is seen to inhibit integrin α5 under profibrotic conditions.^83^ Thus, we speculate that miR-30 functions as a potential anti-fibrotic mediator by translationally reducing the expression of pro-fibrotic proteins.

Another aspect whereby miR-30 family members are linked to pathological remodeling of the heart is related to their implication in cardiomyocyte injury and survival. Down-regulation of miR-30a has been shown to increase p53 level, resulting in enhanced activation of Bax and dynamin-related protein-1.^165^^,^^166^ Subsequently, this promotes mitochondrial impairments and activation of proapoptotic and necrotic pathways, leading to cell death.^117^^,^^165^ This same study suggested that miR-30a is key in mediating the effect of triiodothyronine which is known to hinder maladaptive ventricular remodeling.^167^ Moreover, miR-30-mediated improvement of cardiomyocyte apoptosis involves the up-regulation of the PI3K/AKT signaling pathway via suppression of PTEN expression and the subsequent improvement of cardiomyocyte apoptosis.^168^ In addition, the mitogen-associate protein kinase 4 was established as a direct target of miR-30d and has been shown to mediate its protective effects on cardiomyocytes, especially, through amelioration of cardiomyocyte apoptosis, a key contributor of adverse CR.^83^

## Conclusions and future perspectives

In summary, a growing body of data consistently supports the multiple beneficial effects of SGLT2 inhibition on the heart, kidney, and other physiological functions.^20^^,^^48^^,^^63^ Most likely, these salutary outcomes tend to be a class effect. In particular, their direct actions on CR and endothelial dysfunction are clinically significant and may provide solid clues to explaining the overall outcomes in patients with diabetes. Besides this, dysregulation of the miR-30 family in these pathologic conditions along with their established effects in the endothelium and myocardium are evocating their protective actions against endothelial dysfunction and CR.^112^^,^^115^^,^^162^ Therefore, the coordinated actions of miR-30 and SGLT2is are promising in the understanding of the mechanisms regulating the pathophysiology of these dysfunctions. Yet, from a therapeutic point of view, novel approaches investigating the combined effect of miR-30 and SGLT2is should be considered. Despite significant efforts being made recently in this aspect, there is still an unmet need for in-depth research to establish the cross-talk and the interplay between the involved pathways and molecular mechanisms underlying the effect of SGLT2is and miR-30 together.

Based on the current knowledge, it is reasonable to suggest the miR-30 family, particularly miR-30a, miR-30d, and miR-30e as attractive targets for the diagnosis or the treatment of cardiac and renal dysfunctions.

On the other hand, a better understanding of the regulatory role of ncRNA as new mediators of pathological phenotype in cardiovascular diseases and kidney diseases and their emerging role in paracrine signaling and cell–cell communications may provide insights into new therapeutic strategies to improve care and prognosis. Establishing the mechanistic implication of dysregulated microRNAs may have applications for better and safer management of currently approved therapies, including SGLT2is.

## Author contributions

Conceptualization: R.E.F. and L.R.; Methodology: R.E.F., L.R., and A.E.K.; Writing original draft: A.E.K. and S.M.H.; Review & Editing: R.E.F., L.R., & S.M.H.; Supervision: R.E.F.; Funding acquisition: R.E.F. All authors contributed to the article and approved the submitted version.

## Conflict of interests

The authors declare no conflict of interests.

## Funding

This work was funded by the 10.13039/501100016150OCP Foundation and UM6P University.
